# Femtosecond laser-assisted cataract surgery in patients with phakic intraocular lenses and low endothelial cell counts: a case report

**DOI:** 10.1186/s12886-017-0568-2

**Published:** 2017-10-03

**Authors:** Chia-Yi Lee, Shih-Chun Chao, Chi-Chin Sun, Hung-Yu Lin

**Affiliations:** 1 Department of Ophthalmology, Show Chwan Memorial Hospital, No.2, Ln. 530, Sec. 1, Zhongshan Rd., Changhua City, Changhua, 50093 Taiwan; 2 Department of Electrical and Computer Engineering, National Chiao Tung University, Hsinchu, Taiwan; 3 Department of Optometry, Central Taiwan University of Science and Technology, Taichung, Taiwan; 4 Department of Medicine, Chang Gung University, College of Medicine, Taoyuan, Taiwan; 5 Department of Ophthalmology, Chang Gung Memorial Hospital, Keelung, Taiwan; 6 Department of Chinese Medicine, Chang Gung University, College of Medicine, Taoyuan, Taiwan; 7 Institute of Medicine, Chung Shan Medical University, Taichung, Taiwan; 8 Department of Optometry, Chung Shan Medical University, Taichung, Taiwan; 9 Department of Optometry, Yuanpei University of Medical Technology, Hsinchu, Taiwan; 10 222, Mai-Chin Road, Keelung, Taiwan

**Keywords:** Femtosecond laser, Phakic, Intraocular lens, Endothelial cell density, Cataract

## Abstract

**Background:**

Phakic intraocular lens (PIOL) implantation has been used to correct myopia and myopic astigmatism, although corneal decompensation can occur after implantation. Femtosecond laser-assisted cataract surgery (FLACS) has gained in popularity due to its lower postoperative astigmatism and endothelial loss. Herein, we report the use of FLACS in patients who previously received PIOL implantation and have a low corneal endothelial cell count.

**Case presentation:**

Two patients with a previous iris-claw PIOL implantation were enrolled. The preoperative corrected distance visual acuity (CDVA) and diopter sphere (DS) were 20/32 and −0.25 D in patient 1 and 20/32 and −3.00 D in patient 2. Specular microscope examination revealed an endothelial cell density (ECD) of 1532/mm^2^ in patient 1 and 1620/mm^2^ in patient 2. Capsulotomy was performed smoothly using a femtosecond laser. Postoperative CDVA improved in both eyes, with a difference of DS less than 1 D from the preoperative estimation. Specular microscope examination revealed a decreased endothelial cell density (ECD) in patient 2, but no signs of corneal decompensation were detected.

**Conclusions:**

The influence of using PIOL on capsulotomies performed via FLACS, in combination with preoperative refraction calculation, is minimal. A mild decrease in ECD may occur, but there is a low probability of severe corneal decompensation, even in patients with a low endothelial cell count.

## Background

Phakic intraocular lens (PIOL) implantation has been used to correct myopia and myopic astigmatism; this procedure is better than keratorefractive surgeries in patients with refractive errors of greater than 8 diopters (D) [1]. However, the implantation of iris-fixated and iris-claw PIOL rather than using the posterior chamber PIOL may lead to corneal endothelial loss or corneal decompensation, in which case the PIOL must be explanted [2–4]. Femtosecond laser-assisted cataract surgery (FLACS), introduced in 2008, has gained popularity due to its lower resulting postoperative astigmatism and endothelial loss [5, 6]. Furthermore, the capsulorhexis created by FLACS is more circular and optimally sized compared to conventional capsulorhexis [7]. To date, only two studies have evaluated the feasibility of FLACS in patients with normal corneal endothelial cell count who have received PIOL implantation [8, 9]. Herein, we report results obtained using FLACS in two patients with a low corneal endothelial cell count who had previously received PIOL implantation.

## Case presentation

**Fig. 1:**
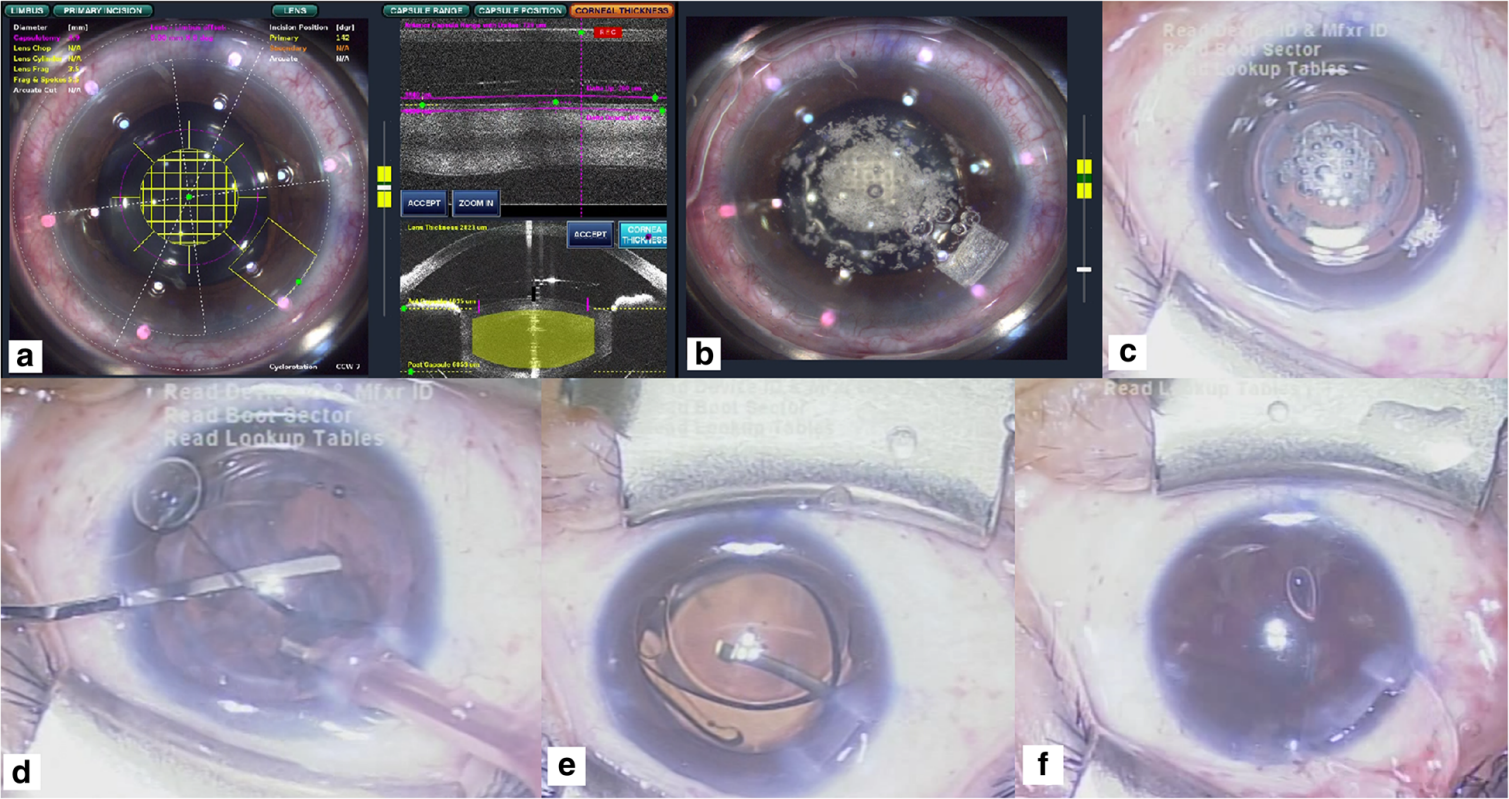
Femtosecond laser-assisted cataract surgery in Patient 1 (with iris-claw phakic intraocular lens implantation). **a** Scheduling of capsulotomy, lens fragmentation and corneal incision positioning in the imaging system. **b** Completion of capsulotomy, lens fragmentation and corneal incision positioning via femtosecond laser. **c** External eye appearance prior to cataract surgery and removal of the phakic intraocular lens. **d** Phacoemulsification and aspiration under the phakic intraocular lens. **e** Implantation of a new multifocal intraocular lens. **f** Extraction of the phakic intraocular lens after widening of the corneal incision

Case 1 is a 41-year-old Taiwanese female who presented with bilateral high myopia, measured at −13.25 D in the right eye. An iris-claw PIOL (Artisan; Ophtec BV, Groningen, The Netherlands) was implanted in May 2006; this preserved visual acuity at approximately 20/25. A right eye nuclear sclerosis cataract was diagnosed in this patient, who had complaints of progressively blurred vision. On examination, the corrected distance visual acuity (CDVA) was preserved at 20/32, with a diopter sphere (DS) of −0.25 D. Specular microscope examination revealed a corneal endothelial cell density (ECD) of 1532/mm^2^. Considering the damage that would have been caused by a second surgery using conventional techniques, PIOL removal concurrent with FLACS was scheduled for the patient. The preoperative estimation of the remaining refractive error was −0.87D via IOLMaster (IOLMaster V.5.4, Carl Zeiss, Oberkochen, Germany). Using the normal mode of the LenSx system (Alcon Inc., Fort Worth, TX, USA) (Fig. 1a), a 4.9 mm capsulotomy (7.00 μJ) and a 2.2 mm corneal incision were made (Fig. 1b). The capsulotomy appearance was good, and cavitation bubbles were restricted below the PIOL after laser fragmentation (Fig. 1c). Nucleus materials were removed by the stop and chop technique using a phacoemulsification device (Centurion, Alcon Inc., Fort Worth, TX) (Fig. 1d). The effective phacoemulsification time was 1.45 s, with a phaco power of torsional mode of 20% to 60% and an irrigation level of 40 mmHg. The multifocal intraocular lens (IOL) (Restor, Alcon) was implanted smoothly into the capsular bag, with no intraoperative complications (Fig. 1e). The PIOL was then removed with forceps after widening the corneal incision to 5.0 mm (Fig. 1f) and the incisional wound was sutured with two stiches. Two weeks postoperatively, the CDVA of the right eye reached 20/20, with a DS of −0.75 D. A specular microscope examination performed on the same day revealed a stable ECD of 1531/mm^2^.

**Fig. 2:**
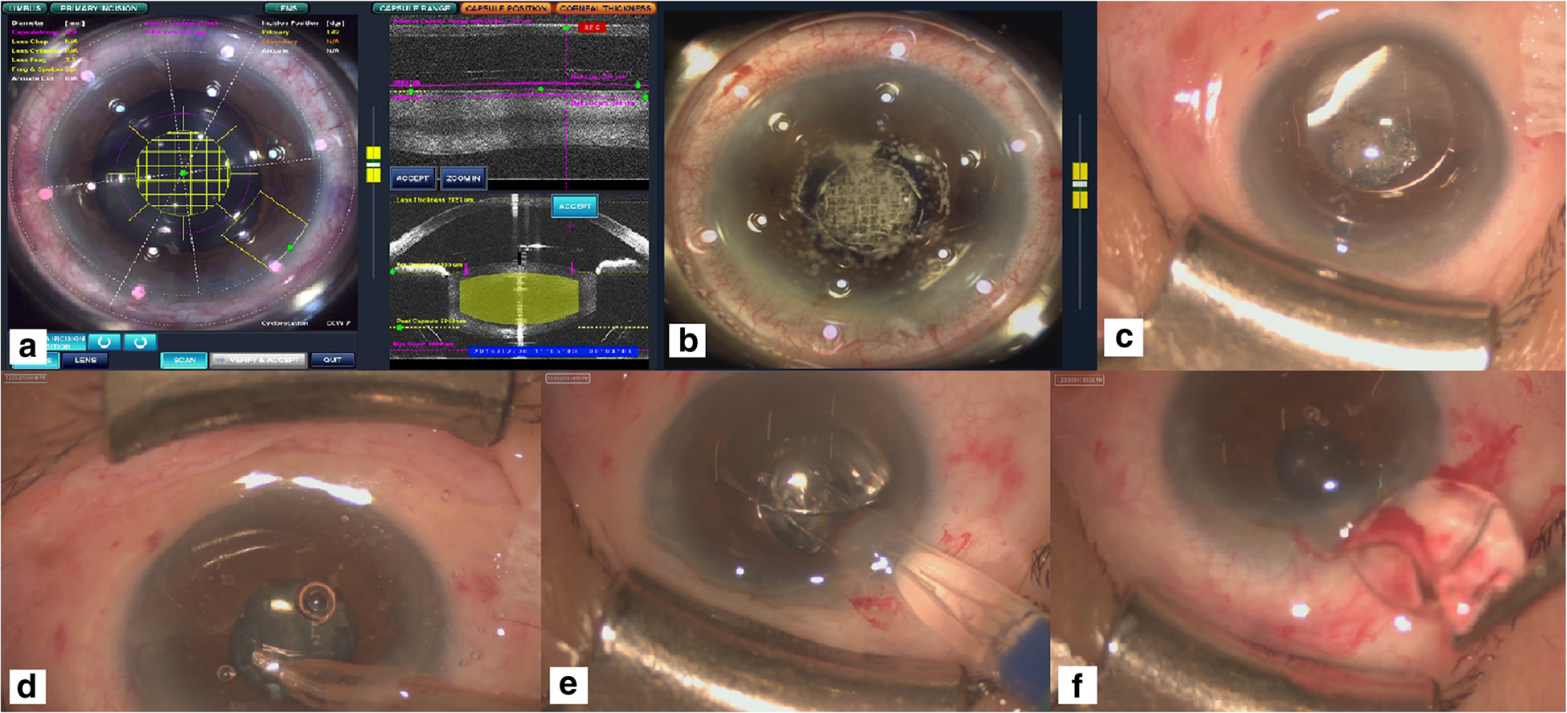
Femtosecond laser-assisted cataract surgery in Patient 2 (with phakic intraocular lens implantation). **a** Scheduling of capsulotomy, lens fragmentation and corneal incision positioning in the image system. **b** Completion of capsulotomy, lens fragmentation and corneal incision positioning via femtosecond laser. **c** External eye appearance prior to cataract surgery and removal of the phakic intraocular lens. **d** Phacoemulsification and aspiration under the phakic intraocular lens. **e** Implantation of the new aspheric intraocular lens. **f** Extraction of the phakic intraocular lens after widening of the corneal incision

Case 2 is a 55-year-old Taiwanese female who received bilateral iris-claw PIOL (Artisan; Ophtec BV, Groningen, The Netherlands) implantation in 2010 due to high myopia (10 D), presenting at the time of examination with right blurred vision for more than 1 year. On examination, CDVA measured 20/32 in the right eye, and DS was −3.00. A right eye nuclear sclerosis cataract was found using a silt-lamp biomicroscope, and a specular microscope examination revealed a decreased ECD of 1620/mm^2^. Since corneal decompensation may occur after two-step conventional surgery, combined surgery was recommended; the patient agreed to undergo the surgery. The estimated refractive error was −0.02 D using the EyeSuit system (EyeSuit™IOL V3.1.0, Haag-Streit Diagnostics). Using the same mode of the LenSx system (Fig. 2a), a 4.9 mm capsulotomy was made (7.00 μJ) with lens fragmentation (Fig. 2b). A laser-created corneal incision was not performed because the position of the PIOL required an adjustment of the incision site. The majority of cavitation bubbles also remained beneath the PIOL until the end of fragmentation (Fig. 2c, d). After the creation of a 2.2 mm manual corneal incision, the stop and chop technique was performed to remove the nucleus using the Centurion device with the following parameters: an effective phacoemulsification time of 1.71 s, phaco power of torsional mode (20% to 60%) and an irrigation level of 40 mmHg. The IOL (Aurium, Medennium) was implanted successfully (Fig. 2e), and the corneal incision was expanded to 5.0 mm to extract the PIOL (Fig. 2f). Then, the incisional wound was closed with two stiches. The follow-up visit 1 month after surgery showed a CDVA of 20/25 and a DS of −0.25 D in the right eye. A specular microscope examination revealed a decreased ECD level of 1044/mm^2^, but the central corneal thickness showed a value of 580 μm, which was similar to the preoperative value of 520 μm. The CDVA of the right eye had improved to 20/20 at the last visit, 10 months after surgery.

## Discussion

In the current study, the feasibility of using FLACS in patients who have previously received PIOL is demonstrated. The results are similar to previous case experiences [8, 9]. In previous studies, cases with PIOL were thought to be more challenging than conventional cases needing manual adjustment [8], and incomplete fragmentation or microadhesions were not uncommon [8, 9]. However, our experience demonstrates that the technical requirements are similar in patients with PIOL because modifications were not made and no capsulotomy tags were found intraoperatively. A previous in vitro study revealed that the IOL can be transected by using the minimum laser energy of 1 μJ [10]. This indicates that PIOL produces a small decrease in laser power.

Even with the innovation of techniques such as torsional ultrasound and viscoelastic devices, cataract surgery increases the risks of corneal decompensation in patients with a low endothelial cell count [11]. Moreover, although FLACS can effectively retard endothelial loss by reducing the effective phacoemulsification time, which correlates with the degree of endothelial damage [5, 6], laser-assisted corneal incision can also damage the endothelium [6]. In the current study, the endothelial cell counts were low for both eyes before surgery compared to an ECD of approximately 2900 cells/mm^2^ in the normal Chinese population [12], and the ECD decreased after the surgery, but there were no signs of corneal decompensation. The combined surgery avoids endothelial damage from double surgery. Further, we removed the iris-claw IOL as the last step for several reasons. First, the PIOL can serve as a barrier to prevent damage to the corneal endothelium resulting from the ultrasound shockwave and mechanical impact. Second, both PIOLs were old products, which required a large incision to extract; late extraction can provide a more stable intraoperative corneal and anterior chamber structure. Nonetheless, synechiae between the long-term implanted PIOL and iris may lead to a hemorrhage when removing the PIOL. If PIOL extraction is done as the last step, the newly implanted IOL can block the hemorrhage, thus making the hemorrhage easier to aspirate. This is a new surgical approach, i.e., using FLACS concurrent with PIOL removal, and it may be applied in certain patients with a low endothelial cell count by using proper management. However, the prominent decline of the ECD in patient 2 makes it clear that there should be further investigation on the optimum threshold for ECD at which FLACS could be avoided.

In previous experience, the refractive outcomes when using FLACS were similar to those obtained conventional methods [5]. The refractive outcome in the current study is satisfactory, with little residual astigmatism (less than 0.75 diopters cylinder and precise DS estimation), despite the influence of the PIOL on the IOL power calculation. To prevent PIOL from interfering in the total refractive power, we used only the corneal refractive power as a reference for refractive calculation. In addition, we recommend that the refractive power of IOLs should be precisely selected in patients who have PIOL due to a high refractive error and because both aspheric and multifocal IOLs have been proven to provide acceptable outcomes in such circumstances.

## Conclusion

In conclusion, the negative influence of the existing PIOL on capsulotomies performed via FLACS, in combination with preoperative refraction calculation, may be minimal. A decrease in ECD could occur in patients with a low endothelial cell count, but severe corneal decompensation does not always develop. However, further large-scale prospective studies are warranted to investigate the safety threshold of FLACS concurrent with PIOL removal in patients with a low endothelial cell count.

## Abbreviation

CDVA: Corrected distance visual acuity
D: Diopter
DS: Diopter sphere
ECD: Endothelial cell density
FLACS: Femtosecond laser-assisted cataract surgery
IOL: Intraocular lens
PIOL: Phakic intraocular lens
